# Multi-Exergames to Set Targets and Supplement the Intensified Conventional Balance Training in Patients With Stroke: A Randomized Pilot Trial

**DOI:** 10.3389/fpsyg.2020.00572

**Published:** 2020-04-02

**Authors:** Imre Cikajlo, Marko Rudolf, Renato Mainetti, Nunzio Alberto Borghese

**Affiliations:** ^1^Research and Development Unit, University Rehabilitation Institute, Republic of Slovenia, Ljubljana, Slovenia; ^2^School of Engineering and Management, University of Nova Gorica, Nova Gorica, Slovenia; ^3^Department of Computer Science, University of Milan, Milan, Italy

**Keywords:** balance, rehabilitation, virtual reality, exergaming, stroke, perception

## Abstract

People who survive a stroke usually suffer movement disorders resulting in involuntary abnormal movements. Intensive and repetitive physiotherapy is often a key to functional restoration of movements. Rehabilitation centers have recently offered balance training supported by exergames in addition to conventional therapy. The primary objective was to investigate different types of balance training (multi-exergaming and conventional) in addition to a conventional 6-week physiotherapy program. Furthermore, we examined the choice of an appropriate exergame to target balance training. We designed a randomized pilot trial. Hospital inpatients with stroke aged 33–65 were recruited and randomized into 2 groups by drawing lots; a control group receiving 1 week of conventional balance training and an exergaming group 1 week of multiple-game exergaming, comprising single leg exercises, weight shifting, balancing and standing up. Center of pressure was monitored for the exergaming group and clinical data were collected (non-blinded assessment) using Four Square Step Test, Timed Up and Go, 10 m Walk Test, Romberg, Sharpened Romberg, Clinical Test for Sensory Interaction in Balance in both groups. Statistical tests were used to find significant (*p* < 0.05) differences and Cohen’s U3 for effect sizes. Recruited participants (20/30) met the inclusion criteria and were randomized; 10 per group. 1 participant of the exergaming group was excluded from center of pressure analysis. Both groups demonstrated substantively and statistically significant improvements of functional balance, in particular the exergaming group (FSST *p* = 0.009, U3 = 0.9 and 10 MWT *p* = 0.008, U3 = 0.9). However, significant differences between the groups were found in tests with eyes closed, Sharpened Romberg test (*p* = 0.05) and standing on the right leg (*p* = 0.035). The center of pressure area decreased up to 20% for the exergaming group. Both types of additional balance training demonstrated comparable outcomes, however, the multi-exergaming could target specific motor control disorders by the selection of exergames according to Gentile’s taxonomy. We may not prioritize exergaming due to the low statistical power of clinical outcomes. However, exergaming enables independent balance training, which is feasible without strenuous physiotherapy and may thus be crucial for future home or telerehabilitation services.

**Clinical Trial Registration:** www.clinicaltrials.gov/, identifier NCT03282968.

## Introduction

Cerebrovascular injury or stroke is a consequence of interrupted blood supply in some parts of the brain due to a blood spill or block in a blood vessel due to clot. People who have experienced a stroke are affected cognitively and physically, and deprived of everyday functions. However, nearly 80% of survivors from approximately 15 million strokes worldwide each year (WHO) suffer impaired motor function and are in need for comprehensive motor rehabilitation to re-enable their everyday life routine (Tyson et al., 2006). Intensive exercises are required for successful motor rehabilitation (Kwakkel, 2009) which is often limited due to time constraints and lack of personnel even in the best rehabilitation clinics. A temporary solution is to prepare the patients for an additional home exercise, which is in most cases unsuccessful due to lack of motivation. These patients rarely meet their prescribed home rehabilitation regimens (Lang et al., 2009) and are advised to visit an outpatient clinic. Clinical studies suggested at least 3 h of timed exercises per week (Foley et al., 2003). The authors agree that the duration and intensity of exercises are crucial for optimal rehabilitation outcomes, regardless of whether rehabilitation is carried out in a clinical setting or at home.

Both rehabilitation environments share one crucial element, i.e., patient motivation. Several researchers have developed rehabilitation games, also as virtual reality, to increase the motivation of patients to more intensively engage in motor rehabilitation (Goršič et al., 2017a). The main focus of rehabilitation games may be to attract patients’ attention and motivate them to achieve a higher score, while virtual reality based rehabilitation offers interactive simulated environments. The users are engaged by these environments, which may be similar to the real world or imaginary objects and events. Rehabilitation games are often controlled by sensors, most recently contactless motion tracking sensors (Microsoft Kinect, Leapmotion, etc.) or force plates (Wii Balance Board, AMTI, etc.) that also serve as assessment devices. Patients with severe stroke may require the assistance of a passive or active rehabilitation robot providing upper limb (Krebs et al., 2004; Oblak et al., 2010) or lower limb support (Chen et al., 2002; Dundar et al., 2014; Meuleman et al., 2016).

Balance and posture are considered complex motor and cognitive functions dependent on various factors, such as muscle strength, mobility, and sensory information from the musculoskeletal system, visual and vestibular systems. Cognitive (Kannan et al., 2019) and emotional factors should also not be neglected (Massion, 1992). Clinical findings emphasize the importance of a large number of regular repetitions of functional movements to achieve neuroplastic changes in the brain, which result in improvement of motor functions (Kwakkel, 2009; Lang et al., 2009). However, conventional physiotherapy often cannot provide a large number of repetitions due to the limited time available for each patient, the limited number of skilled professionals or other related organizational problems (Foley et al., 2003; Langhorne et al., 2009). During the last decade, researchers have proposed exergaming and virtual reality (VR) supported rehabilitation as a tool.

A literature review has provided a long list of publications emphasizing the importance and advantages of using VR and exergames as rehabilitation tools. VR and exergames provide a variety of options for eliciting the maximum voluntary activity of the patient, such as accurate task repetitions, frequency, intensity, change of virtual environments and gradual increase of task complexity (Jack et al., 2001). Patients may practice simulated daily activities (e.g., crossing the road) without continuous supervision by a therapist. Laver et al. (2017) carried out an extensive review concluding that the use of VR by patients with cerebrovascular injuries can be an effective rehabilitation method, especially as an addition to conventional physiotherapy. The approach can prolong the time available for therapy and improve day-to-day performance, as compared with the standard treatment of daily upper limb activities by patients with stroke. Corbetta et al. (2015) reported that substitution of a specific part of conventional rehabilitation with VR can elicit improvements in gait, balance and mobility in persons with stroke. However, most of the reports highlighted that further clinical evidence is required to justify the superiority of either approach (Willaert et al., 2020).

Previous studies have demonstrated that implementation of rehabilitation games into the process of physiotherapy can provide effective or good clinical experience, as well as scientific clinical evidence (Corbetta et al., 2015). Most of the studies were intended for controlled clinical settings in which the entire process was conducted by a medical professional, often a physiotherapist. In the proposed multi-exergaming approach, the role of the physiotherapist would be to supervise and consult on the process. The aim of the randomized pilot trial was to test whether the developed multi-exergame improves balance compared to intensified conventional balance training and strengthening. We hypothesized that using specific types of rehabilitation games based on Gentile’s taxonomy would help to target specific neuromotor control exercises.

## Materials and Methods

### Rehabilitation System

**FIGURE 1:**
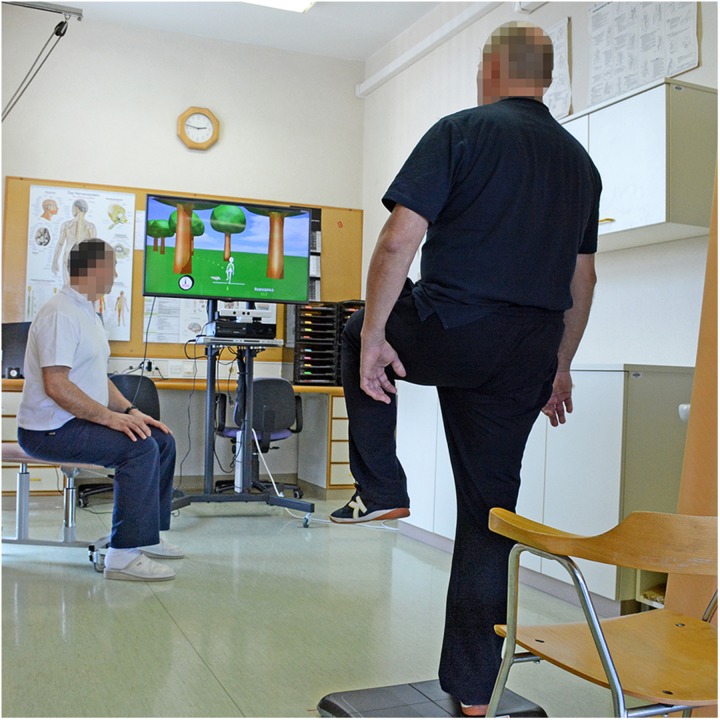
Setup for the exergaming. The participants were standing on the Wii Balance board and played 3 different games. MS Kinect camera tracked the participants’ movements.

The rehabilitation system Rehabilitation Wayout in Responsive Home Environments - REWIRE (Pirovano et al., 2014, 2016) consisted of a large LCD screen (42″), a computer, a Microsoft Kinect^TM^ camera (RGB camera has a standard resolution of 640 × 480 at 30 FPS up to 1024^∗^768 at 12 FPS.), Wii Balance Board (Nintendo Inc., Redmond, WA, United States) and 6 m^2^ of dedicated space. The Microsoft Kinect^TM^ camera was mounted above or under the LCD to capture the entire space dedicated for exergaming. The participant was standing on a Wii Balance Board (WBB) in front of the LCD at a distance of about 2 m (Figure 1). The feet and the legs were held apart as in normal upright posture. After the start of the REWIRE platform, the system was configured and the participant/physiotherapist was able to choose a game and set a level of difficulty and duration. The rehabilitation games were based on Gentile’s Motor Skills Taxonomy (Wüest et al., 2014b), and the “Living on the Farm” theme, providing a calm and relaxing atmosphere for patients. The system enables five levels of difficulty and provides visual and auditory feedback about correct posture.

The exergames were designed to follow a modified Gentile’s taxonomy of motor skill (Magill, 2004; Wüest et al., 2014a). We chose three games suitable for balance, posture and weight shift according to the taxonomy, i.e., 3A–4A body stability without object manipulation and 3C–4C body transport without object manipulation.

**FIGURE 2:**
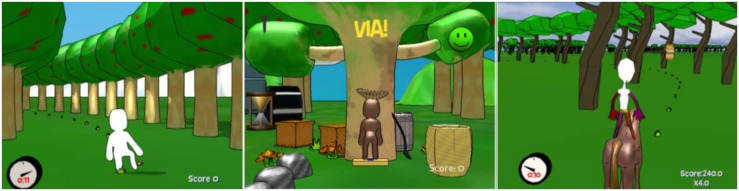
All games were intended for balance and posture, but Animal Hurdler game (1) also for weight shift and single leg standing, FruitCatcher game (2) also for weight shift and steps and The Horse Runner game (3) also for sit-to-stand exercise.

The Animal Hurdler game (Figure 2): This exergame targeted balance and posture, weight shift and standing on a single leg. The participant had to step over small creatures approaching the virtual participant (avatar) by raising a foot. The duration was set to 3 min at difficulty level 3, with definition of the required height for raising the legs (distance of the foot from the ground).

The Fruit Catcher game (Figure 2): This exergame targeted balance and posture, weight shift and stepping. The goal of the game was to catch fruits and avoid chocolate eggs falling from the top of the tree. The virtual participant stood below the tree with a basket on the head and moved the body laterally to catch the fruits falling from the tree. The participant scored when fruit fell into the basket. The fruits fell from different positions on the branches in the horizontal axis. The duration was set to 3 min at difficulty level 3, with definition of the distances for stepping or weight shifting (distance of the fruit from the center).

The Horse Runner game (Figure 2): this exergame targeted balance and posture, and muscle strengthening with a sit-to-stand exercise. The virtual participant sat on a horse that was running in the woods. The horse progressed forward automatically, determining the pace of the exergame. By doing squats the participant could slow down or speed up the horse and avoid hitting branches with the avatar’s head; standing up would make the horse run faster by which the participant received bonus scores as floating honey jars. The duration was set to 3 min at difficulty level 3, with definition of the number of repetitions per minute.

### Participants

**FIGURE 3:**
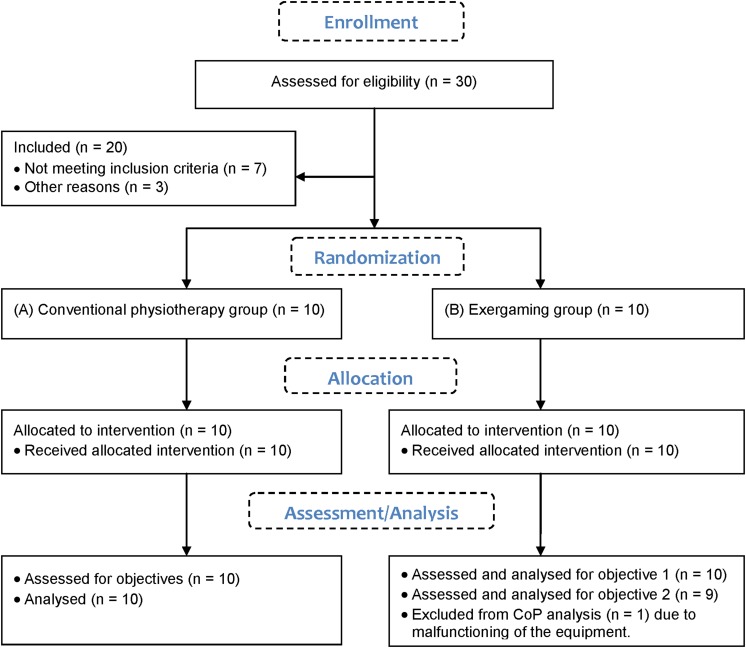
CONSORT flow diagram.

We recruited 30 hospital inpatients with acute and subacute stroke involved in the rehabilitation program of the local hospital, but only 20 were eligible (Figure 3), meeting the inclusion criteria defined by the responsible physician: (1) first ischemic or hemorrhagic stroke, (2) first admission to rehabilitation programs, (3) ability to follow instructions and participate – Mini Mental State Examination (MMSE) > 25, (4) ability to walk independently – minimal FIM^®^ score of 5. A physiotherapist randomized the recruited patients into two groups by drawing lots:

• 10 patients in the conventional physiotherapy (control) group; 9 males and 1 female, 51.8 ± 15.5 years old, 4 had left side and 6 had right side affected after ischemic (6) or hemorrhagic (4) stroke (7.4 months prior to participation in the trial).
• 10 patients in the exergaming group; 6 males and 4 females, 50.3 ± 7.9 years old, 5 had left side and 4 had right side affected and 1 had both sides affected after ischemic (9) or hemorrhagic (1) stroke (4 months prior to participation in the trial).

Patients were subjected to a brief screening test (MMSE) to assess the severity of cognitive impairment with a maximum score of 30. The mean MMSE score for the control group was 27.6 ± 1.8 and for the gaming group 28.6 ± 1.3. Patients were also tested with the FIM^®^ instrument (FIM) to estimate the level of disability and indicate how much assistance was required for the individual to carry out the activities of daily living. Despite the fact that all admitted patients had a FIM score of 6, all experienced problems with static balance, standing on one leg, poor dynamic balance and overall poor performance in functional tests.

### Research Protocol

All participants in the pilot randomized controlled non-blinded trial were part of the same neurotherapeutic treatment at the rehabilitation center. Participants of both groups received 5 additional physiotherapy sessions during 5 consecutive days; one group undertook conventional balance training and strengthening, while the other group undertook exergaming. One day prior to the trial and 1 day after the trial, the participants’ balance and motor abilities were assessed with validated clinical tests.

The exergaming group received regular neurotherapeutic treatment and an additional 15 min of daily targeted exergaming with three different games supported by the REWIRE system. Each participant played three different games for 3 min each, which was just enough time for participants to feel tired, but not exhausted, interspersed by 3 min rest intervals, all games were set to difficulty level 3. The selected games were the weight shifting game Fruit catcher, the squat exercise game Horse runner, and the one leg standing exercise Animal hurdler. The participant stood on the WBB during the exercise, while the movements of the entire body were recorded with the MS Kinect camera (Figure 1). The physiotherapist was present only to supervise the process, ensure safety for the patient and to prevent potential accident.

The control group also received regular neurotherapeutic treatment and an additional 15 min of daily intensive exercise. The manual exercises for the control group were selected specifically to functionally match the exercises performed in the exergames. These exercises consisted of weight shifting between the left and right leg, squats, lifting the legs and balancing at wall bars. The amount of exercise was equivalent to the exergaming in the experimental group (3 times for 3 min with 3 min of resting time). Each participant started with 3 min of weight shifting between the left and right leg, holding the position for 3 s, then shifting weight to the other leg, followed by a 3 min break, then 3 min of squat exercise. Another 3 min rest was followed by alternate lifting of the lower limbs for 3 min. A physiotherapist was present at all times and could offer physical or cognitive assistance, if needed.

### Data Assessment

#### Center of Pressure

Center of pressure (COP) displacement was assessed by the WBB which features four pressure sensors, one at each corner. The WBB measured four vertical forces when the participant was standing with feet apart on the board. The system computed the common COP displacement from provided force data and known board dimensions. The COP displacement data provided by the WBB are perhaps not the most reliable or accurate and are thus not preferable as a clinical diagnostic (Leach et al., 2014), though its repeatability and accuracy are appropriate for exergaming. Besides, the WBB serves as a portable and inexpensive substitute for the force plate in home environments and telerehabilitation applications (Cikajlo et al., 2012).

**FIGURE 4:**
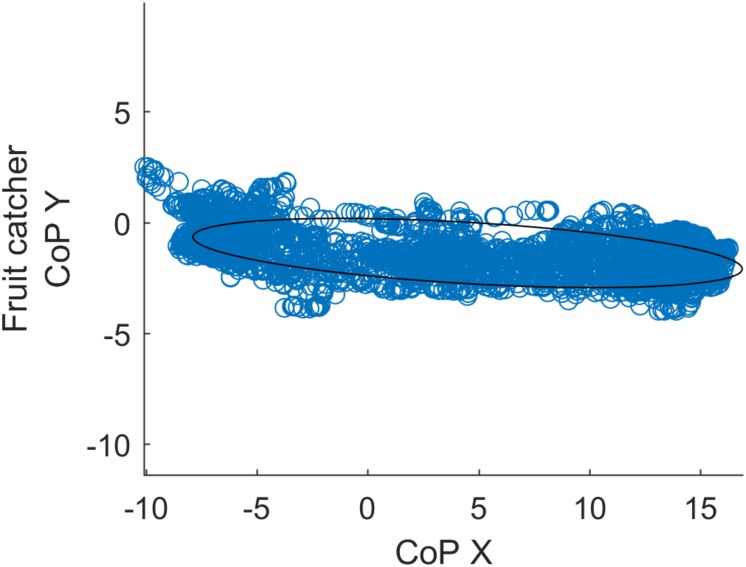
The 95% of the area covered by the ellipse was determined by calculating the eigenvectors.

The COP displacement area depended on the balance/sway performance of the participant. Balance disorders and instability resulted in a wider and larger COP area (Figure 4). The COP area was estimated by the 95% confidence ellipse (Moghadam et al., 2011). The computation was done by subtracting the mean value (Mv) from the set of data (DS) and computing the scale factor (*x*) with the chi-square inverse cumulative distribution function for the given probability (95%):

$$p=F\,\left(x\vee v\right)=\int_{0}^{x}\frac{t^{\left(v-2\right)/2}\,e^{-t/2}}{2^{v/2}\,\Gamma \,\left(v/2\right)}$$

$$x=F^{-1}\,\left(p\vee v\right)$$

where Γ is the Gamma function Γ (*n*) = (*n* − 1)! The covariance matrix (COV) of the mean centered variable (DS-Mv) comprised variances along the diagonal. The COV was scaled by factor *x*. We then computed the eigenvector and eigenvalues of the matrix:

$$D=\left[\begin{array}{cc} \lambda_{1} & \\ & \lambda_{2} \end{array}\right]$$

$$V=\left[\begin{array}{cc} e_{1\,x} & e_{2\,x}\\ e_{1\,y} & e_{2\,y} \end{array}\right]$$

Scaled eigenvectors were applied to project the ellipse (Figure 4) in the parametric form:

$$\left[\begin{array}{c} u_{1}\\ u_{2} \end{array}\right]=\left(V*\sqrt{D}\right)*\left[\begin{array}{c} c\,o\,s\,\emptyset \\ s\,i\,n\,\emptyset \end{array}\right]$$

∅=0⁢…⁢2⁢π

#### Games Score

Total achieved points in each exergame depended on the chosen difficulty level, elapsed time and achieved game score. The difficulty level was set to 3 (medium speed) for all three games. The data were collected during 3 min exercise for each game and each session (day). The game scores were not considered as a measure of participants’ rehabilitation progress, but provided valuable information about the effort and cognitive understanding of the exercise by participants.

#### Clinical Tests

Clinical tests were carried out 1 day prior to the first session and 1 day after the last session, for all participants. Appropriate tests for balance, posture and mobility were selected: Four Step Square Test (FSST) to test dynamic balance while stepping over objects sideways, forward and backward (Whitney et al., 2007), Timed Up and Go (TUG) to assess balance, mobility and fall risk (Ng and Hui-Chan, 2005) and 10 m walk test (MWT) to assess gait performance (Nagano et al., 2015). Additionally, Romberg’s Test (ROM), sharpened Romberg’s Test (sROM), standing on the left leg (STOLL), standing on the right leg (STORL), and the foam and dome test, also called the “Clinical Test for Sensory Interaction in Balance” (CTSIB) were carried out up to 45 s with eyes open and closed. Romberg’s Tests with eyes open evaluated the vision, proprioception and vestibular sensory systems while balancing. The upper limit was set to 45 s. Visual input was removed by closing the eyes, so the participants relied on vestibular and/or proprioceptive sensory systems only. They could not compensate, if there was any severe lesion. Therefore the clinical Romberg test may not detect vestibular problems accurately (Longridge and Mallinson, 2010). The CTSIB was used to quantify the postural control in clinical terms under different conditions (Horn et al., 2015).

### Data Analysis

We examined the COP area in the gaming group during the exergame therapy, requiring adequate postural responses to maintain balance when performing exercises. It was expected that participants with poor balancing abilities would show an evidently larger COP area. The COP area was examined in each individual for five sessions during five consecutive days. We divided the analysis into three parts according to the exergame used and calculated the mean COP area and its standard deviation for each session. On top of that we plotted the regression curve/line and compared the COP area across the three exergames, expecting significant differences due to the different balance/postural tasks in each exergame; standing on one leg, weight shifting, standing up and balancing.

Matlab (MathWorks, Natick MA, United States) was used to extract raw data, calculate COP 95% confidence ellipse and its area. The Matlab Statistical Toolbox was used to calculate the mean; standard deviation of the COP area and repeated measures ANOVA with Tukey HSD *post hoc* test (SPSS 14, IBM Statistics Inc., United States) was used to determine the differences between exergames. Before the analysis data were checked for normality and equality of variances. Additionally, the exergaming scores were statistically analyzed between the sessions for each exergame. The significance level was set to *p* = 0.05.

We carried out separate analyses of the performed clinical tests for the control group and for the gaming group. Data and detailed analysis of the clinical outcomes are available in the Supplementary Material. Data obtained in both groups were tested for normality and equality of variances with Bartlett’s test. In cases where the homogeneity of variances test failed (χ^2^ *p* < 0.05), we used the Man–Whitney *U* Test to determine the differences between the interventions and the Wilcoxon Test for time effect. If the data passed the homogeneity test, we applied a 2-way-ANOVA for intervention and time effect. The significance level was set to *p* = 0.05. Cohen’s U3 index (Cohen, 1977) was used to find effect sizes. The U3 defines the proportion of data from the gaming group that were smaller than the median values of the control group. There was no effect at U3 = 0.5 and maximal at 0 when all gaming group data were above the median of the control group or 1 when all gaming group data were below the median of the control group (effect size: small 0.4/0.6, medium 0.3/0.7, and large 0.2/0.8). The Matlab Statistical Toolbox (MathWorks, Natick MA, United States) with the Measures of Effect Size (MES) Toolbox (Hentschke and Stüttgen, 2011) and GNU PSPP (Free Software Foundation, Inc., Boston, MA, United States) were used for analysis.

## Results

### Exergaming Group

All 10 participants diagnosed with stroke who were randomly assigned to the gaming group accomplished the 5 sessions according to the protocol and completed the assigned clinical tests. The COP analysis excluded missing data from one participant. However, the participant completed all clinical tests and accomplished the game therapy successfully, and was thus not excluded from the trial.

#### Center of Pressure Area

**FIGURE 5:**
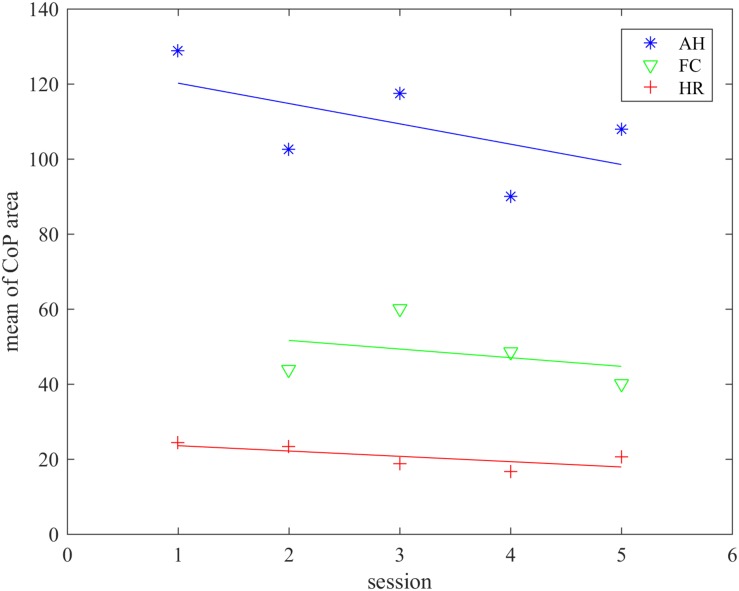
Regression lines of COP area over the sessions demonstrated that there had been an improvement of balance capabilities in all three applied exergames. However, there was no significant difference between the Fruit catcher (FC) and Horse runner (HR) games in terms of COP area (*p* = 0.088), but only between the Animal hurdler (AH) and other two (*p* < 0.003).

Results of the calculated COP area for each exergame per session are presented in Table 1. We removed the data of participant nr. 7 for the FruitCatcher game due to unreliable recordings (however, the participant completed the entire protocol). We calculated the COP area with the ellipse covering 95% of the data assessed. Data were averaged across participants for each exergame and session separately. The mean values and standard deviation values are presented in the Table 1. The decreasing regression line (Figure 5) demonstrated a potentially gradual decrease of COP area and thus a smaller range of motion reflecting improved balance capabilities. The regression line in the Animal hurdler game was the steepest, indicating the greatest impact on the COP and dynamic balance.

**TABLE 1:** Mean COP area during exergaming with three different games (Fruit Catcher, Horse rider, and Animal hurdler) for five consecutive sessions.

	COP area (mean ± standard deviation)				
	Session number				
Exergame	1	2	3	4	5
Fruit catcher	23.84 ± 7.43	43.91 ± 28.93	60.11 ± 34.95	48.59 ± 32.57	40.06 ± 21.95
Horse runner	24.34 ± 6.82	23.33 ± 7.42	18.70 ± 9.71	16.71 ± 11.03	20.56 ± 8.70
Animal hurdler	128.77 ± 66.27	102.54 ± 37.16	117.51 ± 39.78	90.03 ± 21.26	107.91 ± 31.66

*Smaller area of the COP reflects to smaller motion range – in these balance games means improved balance.*

The effects of different games on the COP area during balance training through the sessions were examined. Prior to the repeated measures analysis of variances (ANOVA) a Mauchly’s test of sphericity (Mauchly, 1940) indicated that the assumption of sphericity in the data had been violated, χ^2^ = 45.599, *p* < 10^–4^. Therefore, the Greenhouse–Geisser correction was applied to the degrees of freedom (df) for the *F*-distribution (η = 0.456). The effects within subjects were calculated with repeated measures ANOVA after the corrections had been made. Corrections had no effect on the sum of squares or the *F*, but affected df and *p*-value. We found changes in COP area (Figure 5), but thought the sessions statistically insignificant (*p* = 0.222). Additionally, the interaction effect between time and the exergame was also found insignificant (*p* = 0.114). However, the Tukey HSD *post hoc* test found statistically significant differences between the exergames AnimalHurdler and FruitCatcher, AnimalHurdler, and HorseRider (*p* < 0.003). Statistically insignificant differences were found in the mean COP area between the exergames FruitCatcher and HorseRider (*p* = 0.088).

#### Success in Gaming

The total points achieved in particular exergames were not comparable to each other. Comparison of total points achieved was carried out between the sessions for each particular exergame.

**FIGURE 6:**
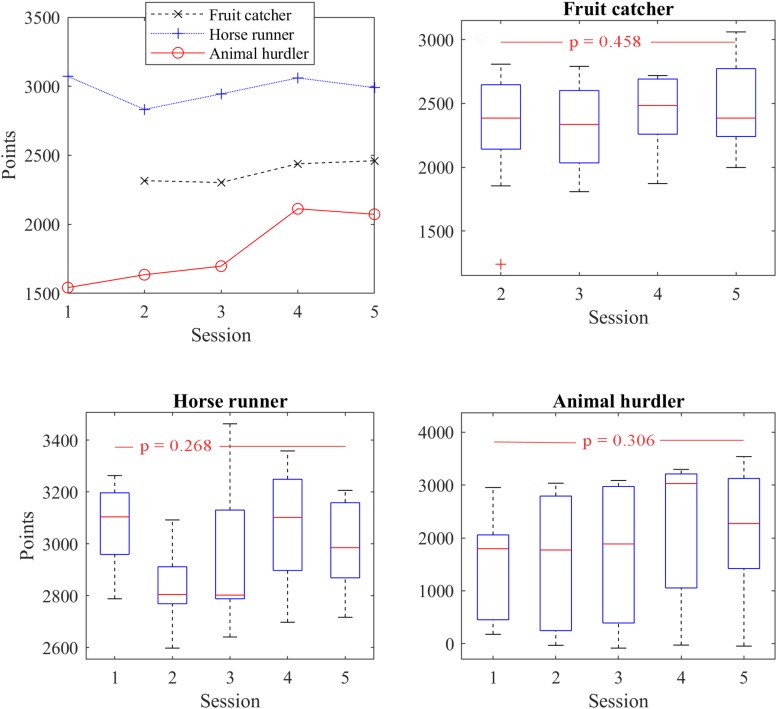
Points achieved in each exergame FruitCatcher (FC), AnimalHurdler (AH), and Horse Runner (HR). Mean values show that participants performed better at the last and pre-last sessions with the FC and AH games. However, the median values and the interquartile ranges revealed that despite of the noticeable progress in AH we had many participants with low score (*p* > 0.05).

The overview of total points achieved in the AnimalHurdler shows that the participants successfully accomplished the game in the last two sessions with 26% higher score (Figure 6). A detailed analysis of the median and interquartile range demonstrated a large variation among results (score range 100–3500, *p* = 0.306), however, the median values showed higher scores at the end of the exergaming. In the 4th session we noticed sample skewness.

In the FruitCatcher exergame the participants achieved 8%, 10% higher mean score at the 4th (mean 2438 points) and 5th session (mean 2460 points, *p* = 0.458), respectively. The analysis of median values (Figure 6), the distribution of the score and interquartile range demonstrated that participants had been able to accomplish the game successfully with equal score even at session 2 (mean 2316 point) and 3 (mean 2303 points). The outcomes of the FruitCatcher game for session 1 were corrupt or missing for three participants, thus we considered the score for this session invalid.

On the other hand, results of the HorseRider exergame show that participants were able to achieve a high score even during session 1 (mean 3071 points) or session 4 (mean 3060 points). The median value of total points dropped at sessions 2 and 3 and the 1.5 × interquartile range demonstrated a large variation in total game score, particularly in session 3. However, the mean values of the HorseRider game slightly dropped at session 2 (mean 2832 points) and was higher in session 3 (mean 2945 points) and session 5 (mean 2990 points, *p* = 0.268).

#### Clinical Outcomes

The balance, postural and mobility tests FSST, TUG and MWT demonstrated functional improvements (Table 2); the FSST failed the Bartlett (χ^2^ = 27.79) homogeneity of variances test and the Wilcoxon’s test found significant functional improvement after the training (mean 13.21 s vs. 10.24 s, *p* = 0.009, U3 = 0.9). Additionally, the test found that 9 participants improved their FSST and only 1 did not. The positive changes of the 10 MWT test (mean 8.76 s vs. 7.14 s, χ^2^ = 23.69) were found significant (*p* = 0.008, U3 = 0.9). Nine participants improved their MWT time, but the MWT for one participant remained unchanged. The Wilcoxon’s test was also used for TUG (χ^2^ = 25.19) and despite the fact that 8 participants improved their TUG and 2 did not, the changes were not statistically significant (mean 9.56 s vs. 8.46 s, *p* = 0.092). However, the effect size was still rather large (*Z* = −1.68, U3 = 0.7).

**TABLE 2:** Results of the clinical tests in exergaming group before and after the additional balance training.

	Gaming group								
	Before		After		Bartlett test	Wilcoxon	ANOVA	Cohen’s	U3 CI
Test	Mean	*SD*	Mean	*SD*	χ^2^ (*p*-value)	*p*-value	*p*-value	U3	[Min max]
FSST	13.21	3.90	10.24	2.44	27.786 (0.000)	0.009*		0.9	[0.3 1]
TUG	9.56	2.69	8.46	2.02	25.187 (0.000)	0.092		0.7	[0.2 1]
10 MWT	8.76	2.48	7.14	1.60	23.689 (0.000)	0.008*		0.9	[0.3 1]
CTSIB eyes open	38.83	13.15	43.52	4.68	Inf (0.000)	0.18		0.5	[0.1 0.5]
CTSIB eyes closed	31.39	19.14	36.69	17.52	0.157 (0.984)		0.341	0.5	[0 0.5]
STOLL eyes open	18.21	19.30	23.74	19.01	0.062 (0.996)		0.575	0.2	[0 0.85]
STOLL eyes closed	1.61	1.18	4.62	5.01	14.122 (0.003)	0.093		0.4	[0.1 0.7]
STORL eyes open	21.53	20.42	23.87	19.40	0.466 (0.926)		0.482	0.4	[0 0.9]
STORL eyes closed	3.92	7.09	2.82	2.02	29.627 (0.00002)	0.674		0.5	[0 0.85]
ROM eyes open	44.33	2.12	43.75	3.95	Inf (0.000)	0.317		0.5	[0.5 0.5]
ROM eyes closed	36.79	13.66	41.49	11.10	5.108 (0.164)		0.046*	0.5	[0 0.5]
sROM eyes open	34.56	17.02	42.11	8.46	6.536 (0.088)		0.369	0.5	[0 0.75]
sROM eyes closed	19.65	19.16	18.96	19.10	2.147 (0.542)		0.969	0.6	[0.1 0.9]

*p < 0.05 statistical significant difference.

CTSIB performance with eyes open improved after the training for two participants and remained the same for the rest and were not sensitive due to the ceiling effect in the majority of participants (mean 38.83 s vs. 43.52 s; χ^2^ = Inf, *p* = 0.18). In the STOLL test with eyes closed (mean 1.61 s vs. 4.62 s, χ^2^ = 14.12, *p* = 0.093) five participants improved their results, three did not and two achieved the same results as prior to exergaming. Four participants were more successful in STORL with eyes closed, four were not and two participants achieved the same results as prior to exergaming, although the changes were not statistically significant (mean 3.92 s vs. 2.82 s, χ^2^ = 29.62, *p* = 0.674).

The outcomes of the ROM test (Table 2) with eyes open could also be neglected due to the ceiling effect of the majority of the participants (mean 44.33 s vs. 43.75 s, χ^2^ = inf, *p* = 0.317). However, changes in the ROM test with eyes closed (mean 36.79 s vs. 41.49 s) were significant over time (ANOVA *p* = 0.046, χ^2^ = 5.11), but there was no effect size due to the ceiling effect (U3 = 0.5). The sROM test with eyes closed indicated minor changes in time (19.65 s vs. 18.96 s, *p* = 0.969, χ^2^ = 2.14, U3 = 0.6). The sROM with eyes opened indicated successful training for three participants, while the other participants achieved the ceiling results (mean 34.56 s vs. 42.11 s, *p* = 0.369, χ^2^ = 6.53).

### Conventional Rehabilitation Group

All 10 participants diagnosed with stroke were randomly assigned to the control group and accomplished additional exercise sessions according to the protocol and accomplished all clinical tests.

#### Clinical Outcomes

Participants in the control group accomplished the TUG and MWT mobility and balance tests (15.18 s vs. 12.17 s and 12.34 s vs. 9.82 s) faster after the training and the results were confirmed as statistically significant (Table 3) by Wilcoxon’s test (χ^2^ = 25.19, *p* = 0.011 and χ^2^ = 23.69, *p* = 0.008, respectively). Nine participants improved their MWT time. Eight participants improved their TUG time, one failed to do so and one remained unchanged. However, the group mean time did not improve in FSST (12.75 s vs. 14.50 s, *p* = 0.575).

**TABLE 3:** Results of the clinical tests in control group before and after the additional balance training.

	Conventional physiotherapy - control group								
	Before		After		Bartlett test	Wilcoxon	ANOVA	Cohen-s	U3 CI
Test	Mean	*SD*	Mean	*SD*	χ^2^ (*p*-value)	*p*-value	*p*-value	U3	[Min max]
FSST	12.75	12.10	14.50	13.78	27.786 (0.00004)	0.575		0.4	[0 0.9]
TUG	15.18	10.20	12.17	6.74	25.187 (0.00001)	0.011*		0.6	[0.2 1]
10 MWT	12.34	8.22	9.82	5.68	23.689 (0.00003)	0.008*		0.6	[0.2 1]
CTSIB eyes open	43.50	4.74	45.00	0.00	*Inf*(0.000)	0.317		0.5	[0.5 0.5]
CTSIB eyes closed	29.13	18.16	34.77	16.81	0.157 (0.984)		0.341	0.3	[0 0.75]
STOLL eyes open	18.82	19.83	20.33	20.57	0.062 (0.995)		0.575	0.5	[0.1 0.8]
STOLL eyes closed	2.20	3.16	2.35	3.40	14.122 (0.003)	0.273		0.5	[0.15 0.9]
STORL eyes open	13.31	16.36	19.33	18.07	0.466 (0.926)		0.482	0.4	[0.05 0.8]
STORL eyes closed	1.29	2.27	1.16	1.29	29.627 (0.00002)	0.753		0.2	[0.05 0.9]
ROM eyes open	45.00	0.00	45.00	0.00	*Inf*(0.000)	–		0.5	[0.5 0.5]
ROM eyes closed	26.46	19.85	40.29	9.94	5.108 (0.164)		0.046*	0.2	[0 0.5]
sROM eyes open	31.90	21.12	34.03	18.07	6.536 (0.088)		0.369	0.5	[0.1 0.8]
sROM eyes closed	8.57	13.36	9.67	13.42	2.147 (0.542)		0.969	0.4	[0.05 0.9]

*p < 0.05 statistical significant difference, A, ANOVA.

On the CTSIB with eyes open (Table 3) the additional training had practically no effect (43.50 s vs. 45 s, *p* = 0.317, U3 = 0.5) due to the ceiling effect of the majority of participants. A larger effect was achieved with eyes closed (29.13 s vs. 34.77 s, *p* = 0.341, U3 = 0.3). Positive but minor changes in STORL with eyes open and STOLL with closed eyes were not statistically significant (Table 3), *p* = 0.753, *p* = 0.273, respectively.

The ROM test with eyes closed demonstrated a large effect size (26.46 s vs. 40.29 s, U3 = 0.2) and significant changes in time (ANOVA *p* = 0.046, χ^2^ = 5.108). The sROM tests with eyes open and closed demonstrated minor changes within the control group (Table 3) and the time effect was statistically insignificant (*p* > 0.369, χ^2^ < 6.53).

### Differences Between the Groups

The differences in age, time since stroke, affected side and MMSE were statistically analyzed for 20 participants randomized into 2 groups. The variances were assumed equal (Leven’s Test) for gender and time since stroke variables, therefore a *t*-test assuming unequal variances was used. We found minor and statistically insignificant differences between the two groups of participants (Table 4).

**TABLE 4:** Analyzing differences between the exergaming group and the conventional physiotherapy control group.

Variable	Control group	Gaming group	Levene’s Test (*F*/*p*)	*T*-test equality of means (*p*-value)
Gender (M/F)	9/1	6/4	12.05/0.03	0.138
Age (mean/*SD*)	51.8/15.48	50.3/7.90	2.19/0.156	0.788
Affected side (L/R/both)	4/6/0	5/4/1	1.45/0.245	1
MMSE	27.6	28.6	1.33/0.263	0.200
Stroke (years)	7.4	4	5.16/0.036	0.235

**FIGURE 7:**
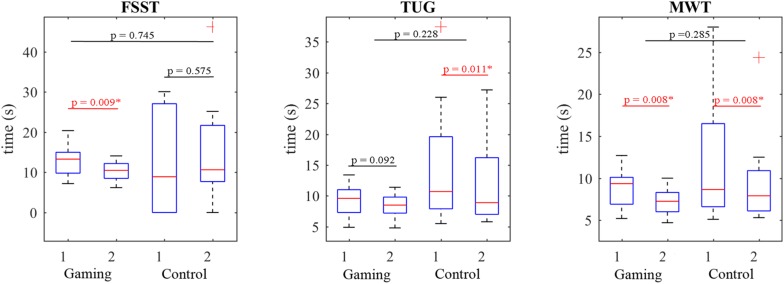
The gaming group (G) demonstrated functional improvements after the training (2-G) in FSST (*p* = 0.009), TUG (*p* = 0.092), and MWT (*p* = 0.008) tests. The control group (C) also improved their TUG and MWT time comparing to the initial results (1-C). Substantive differences were found between the groups in MWT (U3 = 0.7).

Despite the fact that the gaming group demonstrated significant progress in motor and balance tests MWT (*p* = 0.008) and FSST (*p* = 0.009), but not in the gait test TUG (*p* = 0.092), the outcomes were not significantly different (*p* > 0.05, Mann–Whitney *U* Test) from the control-group in any of these tests (Figure 7).

**FIGURE 8:**
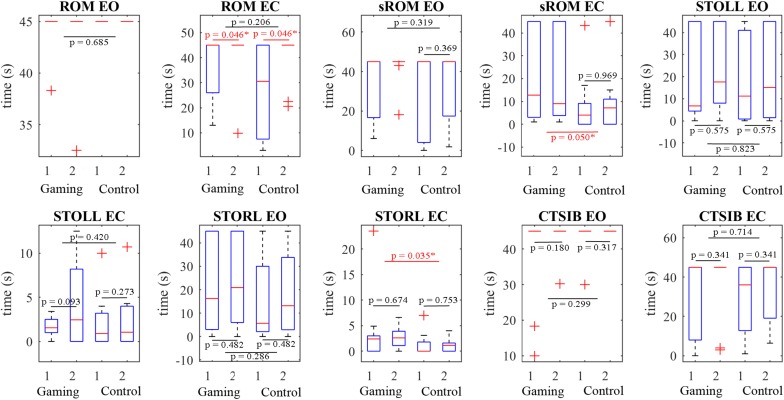
The clinical tests ROM, STOLL, STORL, and CTSIB showed improvements of median score after the training (2), except the sROM with closed eyes for the gaming group (G). However, differences between the control (C) and gaming (G) groups were found only in two tests; STORL EC (*p* = 0.035, U3 = 0.3) and sROM EC (*p* = 0.05, U3 = 0.4).

Standing on the right leg with eyes closed (STORL EC) demonstrated significant differences between the groups (*p* = 0.0345, Mann–Whitney *U* Test, Figure 8), but medium effect size (U3 = 0.3).

Practically no statistically significant differences between the gaming group and control group were found in tests performed with eyes open [ROM EO, sROM EO, STOL(R)L EO, and CTSIB EO]. Mean values between the groups were found to differ in ROM EC and sROM EO tests, but differences in these tests between the gaming and control group were statistically insignificant (ANOVA group effect *p* = 0.206, 0.319, respectively). Statistically significant differences between the participants in gaming and control groups were found only in sROM (*p* = 0.05, χ^2^ = 2.15, ANOVA) and STORL (*p* = 0.035, χ^2^ = 29.63 Mann–Whitney *U* Test) tests, both performed with closed eyes. These tests also demonstrated small to medium effect sizes, U3 = 0.4 and 0.3, respectively (Figure 8).

## Discussion

### Exergaming Group

Participants in the gaming group managed to accomplish the exergames as required by the protocol. The mean COP area calculated from the anterior–posterior and mediolateral displacements have decreased from the 1st to the last session for the 3 applied exergames. The COP area gradually decreased from session to session in particular for the HorseRunner exergame. For the AnimalHurdler exergame the participants’ COP area was rather alternating, though the regression line’s negative slope showed a reduction of COP area size. The exergame FruitCatcher was evidently an easier game to play requiring only medio-lateral movements. The participants’ mean COP area increased in the 2nd session and then gradually decreased until the last session. However, the statistically significant difference of the mean COP area between the AnimalHurdler and HorseRunner suggests that these exergames targeted different gait, balance or posture parameters. While AnimalHurdler was intended for balance and single leg standing, the HorseRunner was designed for practicing balance and standing up (Pirovano et al., 2014). The FruitCatcher was designed for balance, posture and weight transfer, therefore the statistical differences in COP area displacements found with the AnimalHurdler exergame were not surprising. Nevertheless, the FruitCatcher and HorseRunner exergames required the most similar functional balance tasks as shown by the outcomes. These findings may be important for choosing an appropriate exergame when targeting specific balance or gait parameters. We found significant improvement of balance parameters in the exergaming group with FSST, gait parameters with the 10 MWT and positive changes with TUG mobility test, even if statistically non-significant. All participants were able to finish the FSST test that required stepping forward, sideways and backward on the squares in clockwise and counterclockwise directions, even faster than before the exergaming program. This means that they may have improved their dynamic balance performance as the test required loading on the affected side while facing forward. The COP also showed an improvement of participants’ ability to shift the center of mass and maintain stability while stepping. These findings were in line with our expectations and previously published studies suggesting that COP area displacement had been positively correlated to the balance and gait parameters (Lopes et al., 2015). The studies also reported results that were correlated to muscle strength, gait performance, balance, postural control and stability, and their improvement with the reduction of COP area after the rehabilitation/physiotherapy in patients diagnosed with stroke (Paillex and So, 2005), and appeared to be significantly different between persons diagnosed with stroke and healthy persons (Rode et al., 1997).

The attractiveness of the exergames plays an important role in rehabilitation outcomes (Hung et al., 2016) therefore the achieved game score is not negligible information. The achieved mean game scores for FruitCatcher and AnimalHurdler were higher at the end than during the 1st or 2nd session indicating that participants’ effort was still present. The mean score with HorseRunner was rather fluctuating. This information was not necessarily directly related to the improvement of balance abilities, but rather presented an intrinsic motivation factor for the participants (Goršič et al., 2016).

### Conventional Physiotherapy Group

The provided balance training program contributed to the statistically significant improvement of the two mobility tests, 10 MWT and the more comprehensive test requiring standing up, the TUG. A detailed insight into the participant’s data revealed that actually each individual patient improved his/her score in the applied clinical tests or measures were not sensitive due to the ceiling effect. With the exception of one patient who was not able to stand on a single limb with eyes closed and was also not able to finish the FSST. Closing the eyes deprived the participants of visual information. Those with well-preserved proprioception and vestibular sensory system functionalities would have been compensating for vision. But dysfunction of the vestibular system is often present in persons with stroke or brain injury and people suffering from middle cerebral artery stroke show large asymmetrical lateral displacement, responses to postural perturbation are asymmetrical and balance is hindered (Marsden et al., 2005). Therefore, balancing and postural control of the studied patients at baseline mainly relied on proprioceptive control. After the intervention, statistically significant difference of means with the baseline was found using the ROM with eyes closed. Obviously the additional balance training had a positive influence on vestibular system functions, considering that the clinical instrument ROM is intended for vestibular disorders assessment (Gottshall, 2011) and that patients were deprived of visual information.

### Advantages of Exergaming Over Conventional Physiotherapy

In terms of clinical outcome, additional exergaming was equivalent to conventional physiotherapy by the control group. Both approaches demonstrated progress in ROM with closed eyes after the training. This means that both forms of balance training that were added to the rehabilitation program resulted in improvement of vestibular sensory system functionality.

The functional test FSST targeting vestibular disorders (Whitney et al., 2007) evaluation also confirmed our findings. The positive changes in FSST could be related to the additional exergaming based balance training (U3 = 0.9) despite the short, but intensive program as in the control group we found minimal effect size (U3 = 0.4). Blennerhassett and Jayalath (2008) reported that the main changes in FSST were noticed from the baseline to the 2nd week and only minor changes were reported from weeks 2 to 4. The FSST was considered a dynamic balance test that could successfully assess changes during balance training in the stroke population and provide ancillary information about daily living activities. Participants who completed the test in more than 12 s were classified in a group with increased risk for falls (Whitney et al., 2007). According to these criteria both participating groups were considered fallers before the additional balance training. However, only the exergaming group achieved the mean score of 10.24 s, defining them as non-fallers. Besides, the change was considered statistically significant (*p* = 0.009). TUG and MWT both demonstrated clinically proven improvements, but no statistical difference between these approaches was found.

The major advantage of the exergaming approach was the access to the objective, measurable information like the COP. The COP area displacement was reported to be in correlation with clinically proven tests for balance and posture evaluation (Lopes et al., 2015), and furthermore, we found differences between the applied exergames. Therefore an appropriate choice of exergame may help to target specific balance or postural disorder.

The gaming group found the additional balance training challenging according to the achieved score which is in line with the findings of Hung et al. (Hung et al., 2016) who reported the importance of game diversity and fun.

We have demonstrated that exergaming can compare to intensive conventional balance training as an addition to the regular rehabilitation program. Furthermore, valuable results were demonstrated by FSST and tests with closed eyes. We do not expect that virtual reality based rehabilitation is more effective than conventional rehabilitation (Corbetta et al., 2015), but in our opinion we could achieve promising results with the additional balance and mobility training without replacing existing clinical methods and possibly extend the rehabilitation period for a larger stroke population, by enabling them to perform some exercises at home.

### Limitations of the Trial and Future Work

The aim of the trial was to demonstrate that the additional multi-exergaming is comparable with conventional exercises for patients recovering from stroke. We measured the kinematics of the whole body with the camera and used the COP with a low-cost force plate as a bio-feedback for the exergame, while the participants performed the exercises. The COP was not assessed in the control group due to the conventional clinical exercises for balance training that did not use the balance board. Despite the fact that game scores were a good indicator of participants’ motivation/effort, we could also have applied the intrinsic motivation inventory (Goršič et al., 2017b) to both groups.

Clinical tests demonstrated much higher variance than expected for the planned statistical power (0.8). The recruited sample size (30) would have been large enough for the planned objective assessments and statistical power, but unfortunately not all patients were eligible for the trial. Therefore, the small sample size caused violation of the assumption of sphericity (Mauchly’s test) and statistical degrees of freedom were corrected in the trial. Although we found balance training equivalent in both groups as reported by the literature (Corbetta et al., 2015) and rather medium to large effect sizes were found, the small sample size is a limiting factor. Consequently, there is a high risk of bias. Another non-negligible factor could be a lack of motivation, ability to perform required exercises, poor sensibility or muscle tone. Therefore, we suggest a multicenter trial including intrinsic motivation inventory and the use of objective assessment methods for future randomized clinical trials to promote exergaming. Particularly if the proposed tool is intended for objective evaluation of remote (tele)rehabilitation.

## Conclusion

Stroke patients who required minimal assistance while undergoing physiotherapy demonstrated that a regimen of varied exergames was as effective as traditional exercise, to target and improve balance, posture, single leg standing, weight shifting and muscle strength. Selection of specific exergames is important when planning additional treatment to conventional rehabilitation programs. Clinical evaluation with the FSST balance test, the Romberg test with eyes closed and the 10 MWT mobility test, demonstrated significantly positive outcomes. However, the exergaming procedure, contrary to additional conventional exercises, did not require manual handling or intervention by a physiotherapist and thus holds potential for telerehabilitation.

## Data Availability Statement

All datasets generated for this study are included in the article/Supplementary Material.

## Ethics Statement

The study (Approval Number: 10112016) was reviewed and approved by the Ethics Committee of University Rehabilitation Institute, Republic of Slovenia and all participants provided an informed written consent. The procedure was in accordance with the principles of the Declaration of Helsinki on biomedical research on human beings, the provisions of Council of Europe Convention on the Protection of Human Rights and Dignity of the Human Being with regard to the Application of Biology and Medicine (Oviedo Convention) and the principles of Slovenian Code of medical ethics. Written informed consent was obtained from the individual(s) for the publication of any potentially identifiable images or data included in this article.

## Author Contributions

IC led the research, made the analysis, and wrote the main structure of the manuscript. MR carried out the physiotherapy and clinical work/data. RM set up the equipment and contributed to the technical description. NB was responsible for the entire REWIRE project. All authors read and approved the final manuscript.

## Conflict of Interest

The authors declare that the research was conducted in the absence of any commercial or financial relationships that could be construed as a potential conflict of interest.

## Supplementary Material

DATA SHEET S1 Data and detailed analysis of the clinical outcomes.
